# Unveiling the Rare: A Case Report of Rectal Schwannoma in a Neurofibromatosis Type 1 Patient

**DOI:** 10.7759/cureus.82808

**Published:** 2025-04-22

**Authors:** Sai Swarupa Vulasala, Jacob Robinson, Aryan Sharma, Sean Wehry, Omer Mohamedahmed, Dheeraj Gopireddy

**Affiliations:** 1 Diagnostic Radiology, University of Florida College of Medicine – Jacksonville, Jacksonville, USA; 2 Biology, Creekside High School, St. Johns, USA; 3 Pathology and Laboratory Medicine, University of Florida College of Medicine – Jacksonville, Jacksonville, USA

**Keywords:** benign peripheral nerve sheath tumor, neurofibromatosis type 1 (nf-1), rectal schwannoma, schwann cells, schwannoma imaging

## Abstract

Neurofibromatosis type 1 (NF-1) is an autosomal dominant neurocutaneous disorder characterized by skin abnormalities, such as café-au-lait macules and skinfold freckling, as well as peripheral nerve sheath tumors such as neurofibromas, schwannomas, and various other tumors. A 28-year-old man with a history of NF-1 presented to our facility with rectal bleeding. A rectal mass was subsequently discovered on colonoscopy and subsequent imaging. This mass was biopsied with histopathology consistent with a low-grade schwannoma. Schwannomas, while less common in NF-1 than NF-2, do sometimes occur in NF-1 patients, most commonly along the cranial, spinal, or peripheral nerves. These tumors also rarely occur in the gastrointestinal tract and even more rarely within the rectum specifically. Schwannoma must be considered in the differential for any rectal mass discovered in a patient with NF-1, so that the patient can be appropriately managed and treated.

## Introduction

Neurofibromatosis type 1 (NF-1), also known as von Recklinghausen disease, is an autosomal dominant neurocutaneous disorder caused by loss-of-function mutations in the NF-1 gene [1]. Its incidence ranges between one in every 2000 and 3000 newborns and is prevalent among one in every 4000-5000 individuals [1-3]. It is characterized by skin abnormalities, such as café-au-lait macules, skinfold freckling, and neurofibromas, leading to a predisposition to various neoplasms, and is equally common in both men and women. Although benign neurofibromas are the hallmark neoplasms of NF-1, other nerve sheath tumors such as schwannomas can also occur. Schwannomas are benign peripheral nerve sheath tumors which occur secondary to the increased proliferation of Schwann cells. While schwannomas are more commonly associated with NF-2, these can be seldom encountered with NF-1 [4]. They are typically found along the cranial, spinal, or peripheral nerves where there are abundant Schwann cells. Schwannomas in the gastrointestinal tract are rare, constituting approximately 2-6% of all the gastrointestinal mesenchymal tumors (GIMT) with most cases reported in the stomach (83%) or small intestine (12%) [5,6]. In contrast, rectal schwannomas are very uncommon, making these challenging to diagnose. This report presents a case of an anorectal schwannoma in a patient with NF-1, showing a rare manifestation of the disease. The clinical presentation, imaging studies, and histopathological findings in this case show the importance of including schwannomas in the differential diagnosis of rectal masses. Recognizing the rare manifestation of this disease is critical for ensuring the appropriate management and treatment of this condition.

## Case presentation

**Figure 1:**
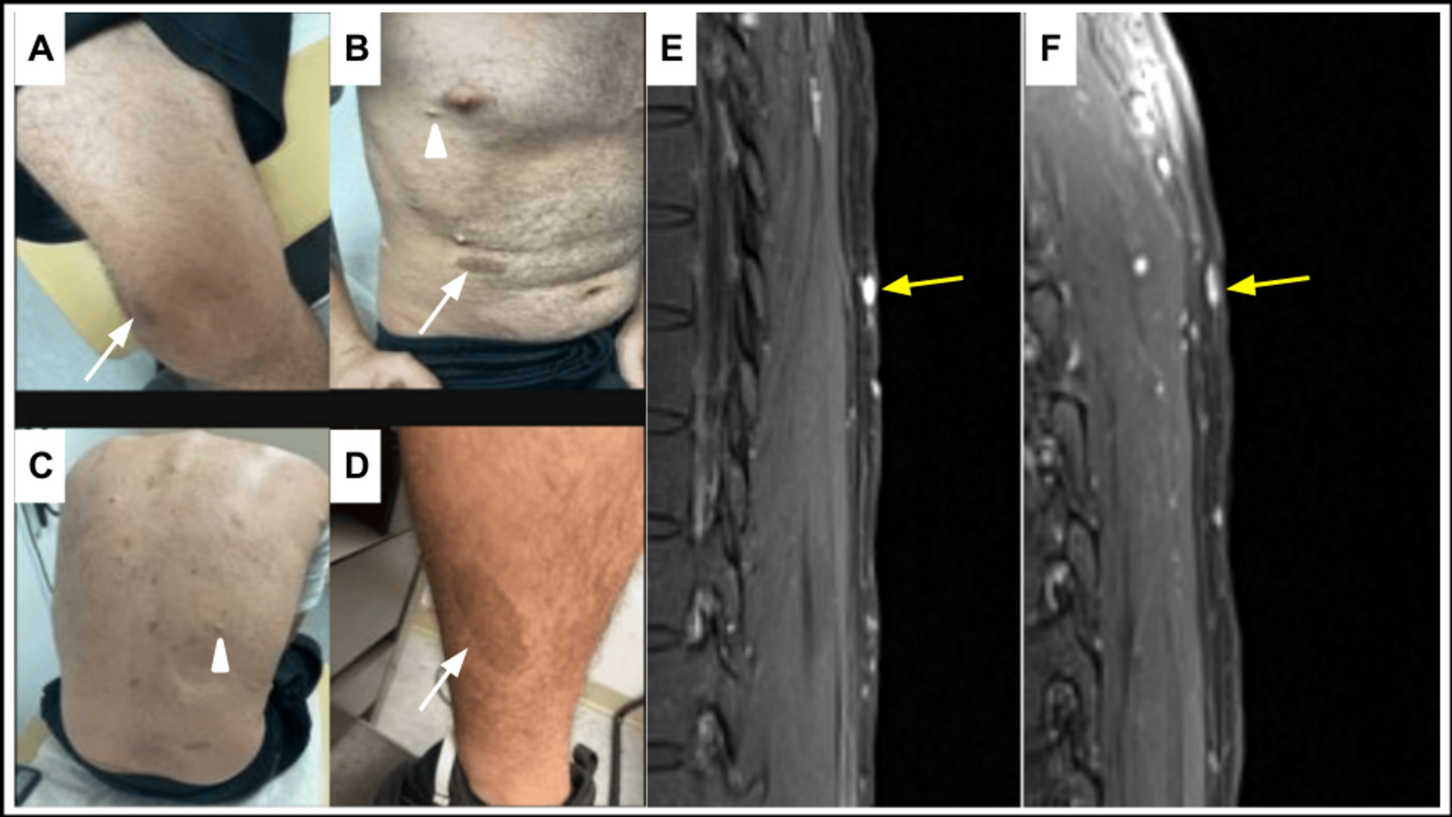
(A-D) Images demonstrate pigmented skin lesions consistent with café-au-lait macules (white arrows). Additionally, there are tiny cutaneous nodules (arrowheads) consistent with cutaneous neurofibromas in this patient. (E) T2-weighted magnetic resonance imaging sequence demonstrates hyperintense subcutaneous lesions (yellow arrow). (F) On gadolinium administration, these lesions demonstrate vivid contrast enhancement (yellow arrow).

A 28-year-old male patient with a medical history of NF-1, spinal stenosis, attention deficit hyperactivity disorder, and intellectual developmental disorder presented with rectal bleeding. He denies experiencing abdominal pain, nausea, vomiting, constipation, fecal incontinence, weight loss, fever, or chills. While the patient has no significant history for smoking or alcohol or drug abuse, he has a family history of unknown cancer in his maternal grandmother and colon cancer in his maternal uncle. Vitals were within normal limits. Physical examination revealed multiple pigmented areas consistent with café-au-lait spots (Figure 1). Numerous tiny cutaneous nodules were noted throughout the patient's body consistent with neurofibromas (Figure 1). 

**Figure 2:**
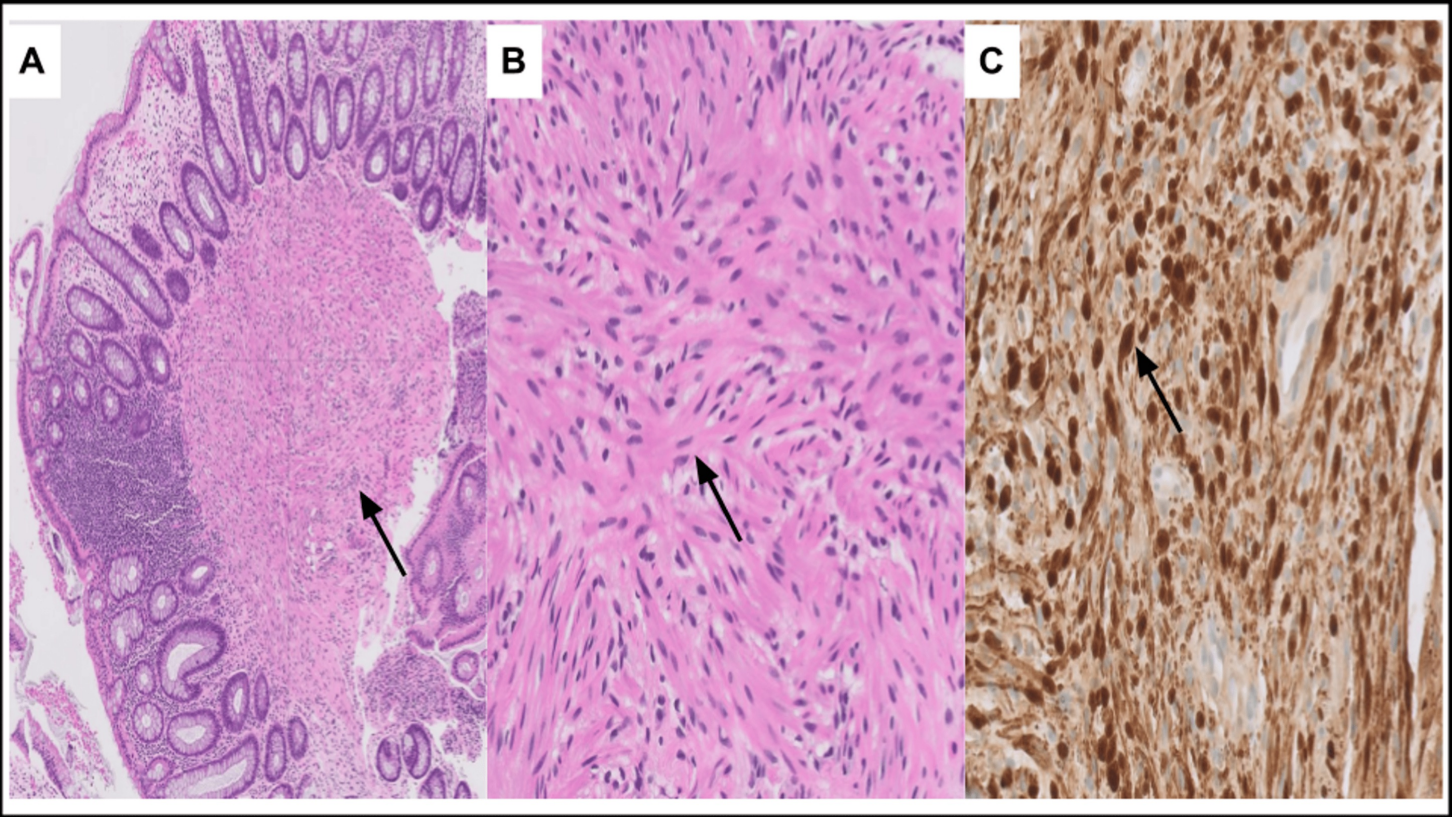
Microscopic images of H&E-stained sections and S-100 immunohistochemical stains of schwannoma. (A) Low-power view (×5) displays non-encapsulated polypoid lesion arising in the muscularis propria (black arrow). (B) High-power view (×20) displays uniform (monomorphic) cells with narrow, elongated, and wavy tapered ends with no overt features of malignancy (no mitosis or atypia identified) (black arrow). (C) SOX-10 immunohistochemical stain shows positive diffuse nuclear staining pattern (black arrow). H&E: hematoxylin and eosin; SOX-10: SRY-box transcription factor 10

The digital rectal exam revealed a firm circumferential rectal mass. On colonoscopy, a submucosal non-obstructing and non-bleeding rectal mass was identified which was 75% circumferential. This mass was biopsied, and the histopathology (Figure 2) showed spindle cell proliferation which were diffusely positive for S-100 and SRY-box transcription factor 10 (SOX-10) immunohistochemical markers. The tissue was negative for epithelial membrane antigen, desmin, chromogranin, and cluster of differentiation 34 (CD34). There was no significant mitotic activity, and the cells showed minimal atypia suggestive of benign neurogenic/nerve sheath proliferation such as schwannoma. The mass was negative for histone H3 lysine 27 (H3K27) trimethylation deletion, a finding which is seen in more aggressive nerve sheath tumors. 

**Figure 3:**
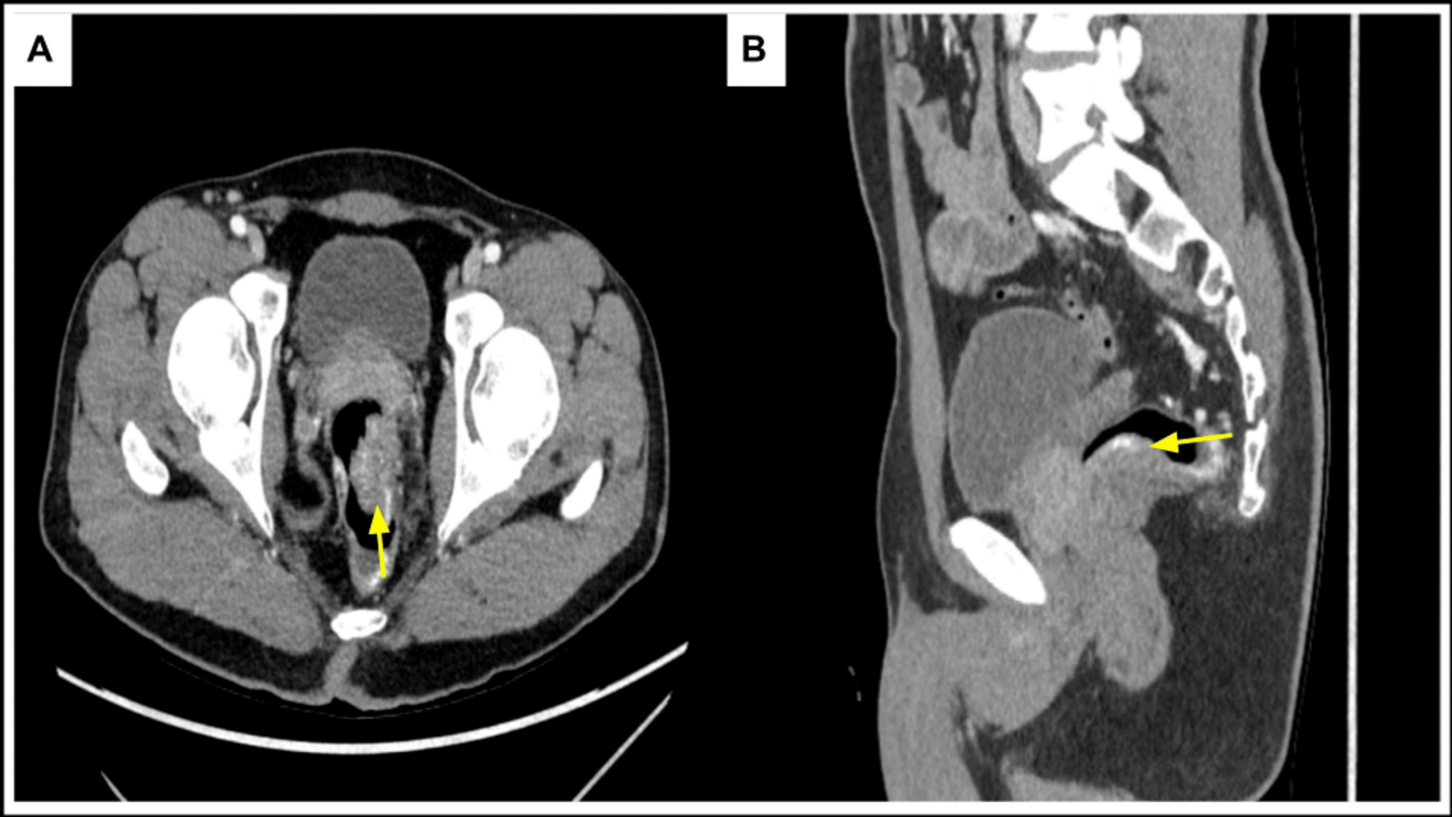
Computed tomography of the pelvis with intravenous contrast on portal venous phase demonstrates a heterogeneous enhancing endoluminal soft tissue lesion (yellow arrow) arising from the rectal wall viewed on axial (A) and sagittal (B) planes. This is consistent with biopsy-proven rectal schwannoma.

**Figure 4:**
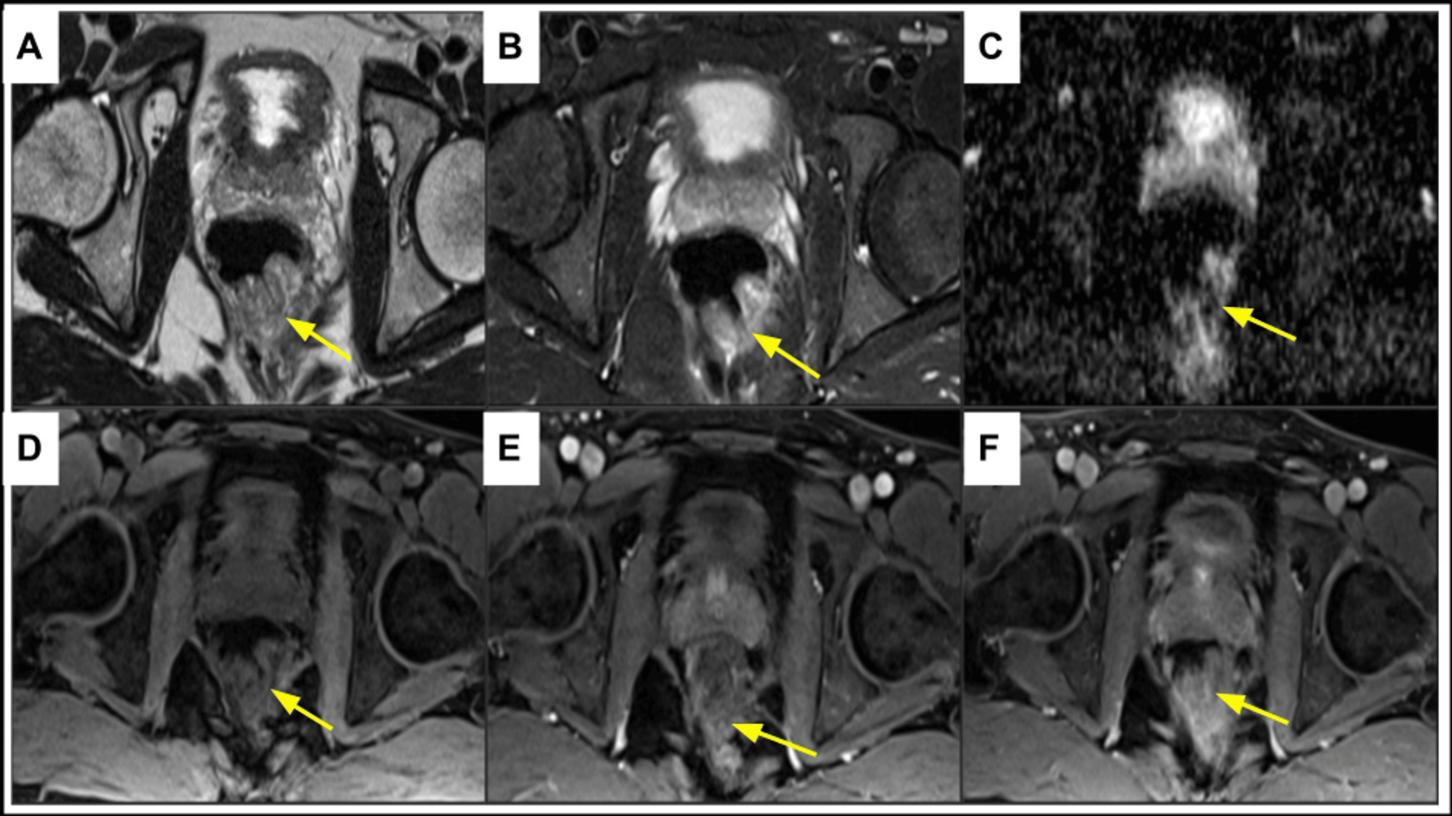
Magnetic resonance imaging of the pelvis demonstrates heterogeneous predominantly hyperintense rectal wall lesion (yellow arrow) on T2-weighted non-fat-saturated (A) and T2-weighted fat-saturated (B) imaging sequences. The lesion demonstrates restriction on diffusion-weighted (C) images. T1-weighted pre-contrast (D), post-gadolinium arterial phase (E), and post-gadolinium delayed phase (F) images demonstrate intense contrast enhancement of the rectal lesion. This lesion is consistent with biopsy-proven rectal schwannoma.

**Figure 5:**
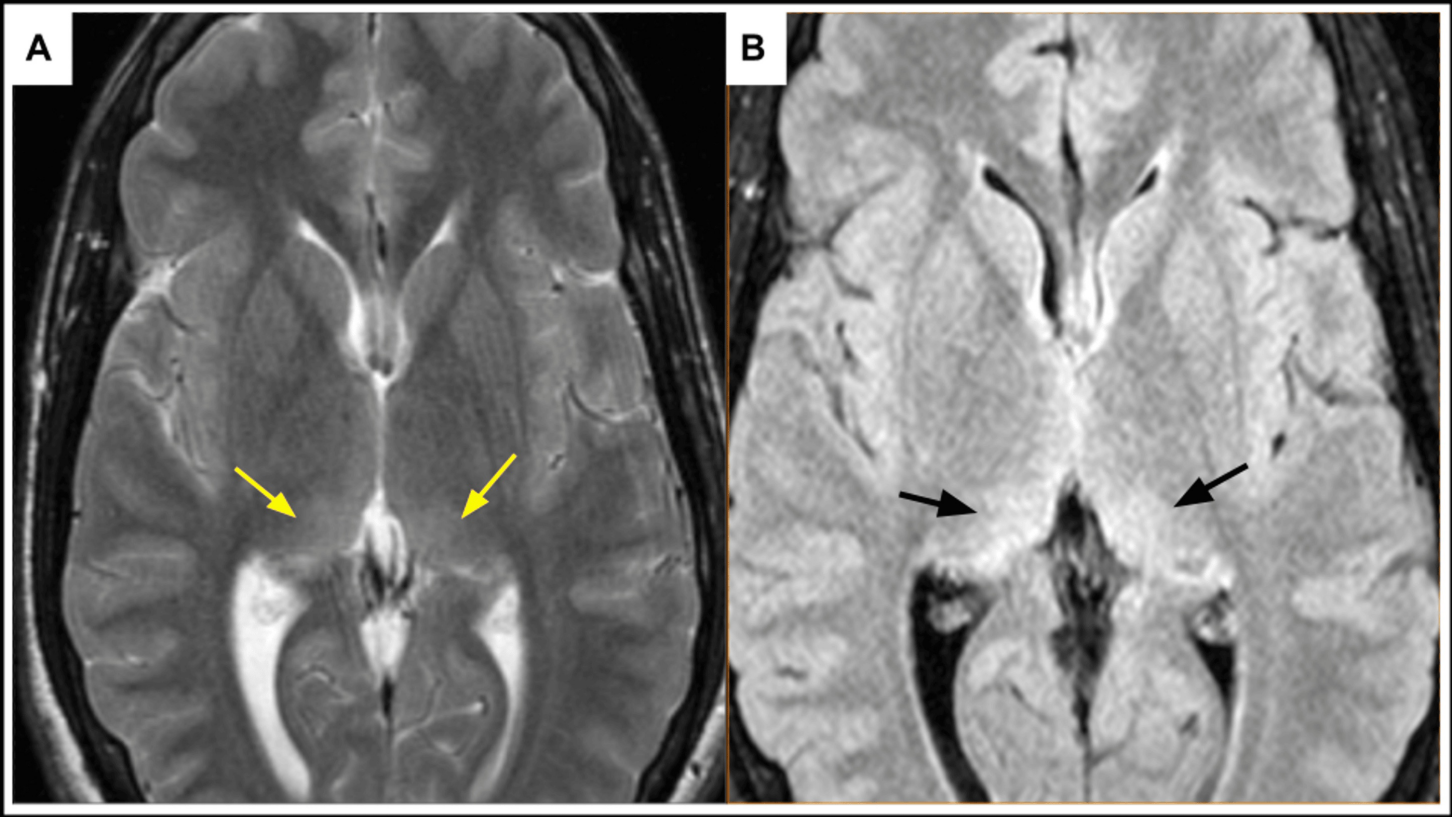
Magnetic resonance imaging of the brain demonstrates FASI within the medial thalami bilaterally on T2-weighted (yellow arrow) (A) and fluid attenuation inversion recovery (black arrow) (B) sequences. FASI: focal areas of signal intensity

Computed tomography (CT) of the abdomen and pelvis with intravenous contrast (Figure 3) demonstrated an endoluminal rectal mass measuring 2 × 3.3 cm with significant vascularity. Further imaging with magnetic resonance imaging (MRI) of the pelvis with and without contrast (Figure 4) revealed a lower rectal polypoid submucosal lesion measuring up to 3.7 cm with the involvement of the anorectal junction. Additionally, the MRI of the brain demonstrated characteristic focal areas of high signal intensity along the bilateral posteromedial thalami (Figure 5). The patient is currently scheduled for surgical management aiming for negative tumor margins. The goal of surgery is to ensure that entire tumor tissue is removed to minimize the risk for local recurrence. 

## Discussion

**Figure 6:**
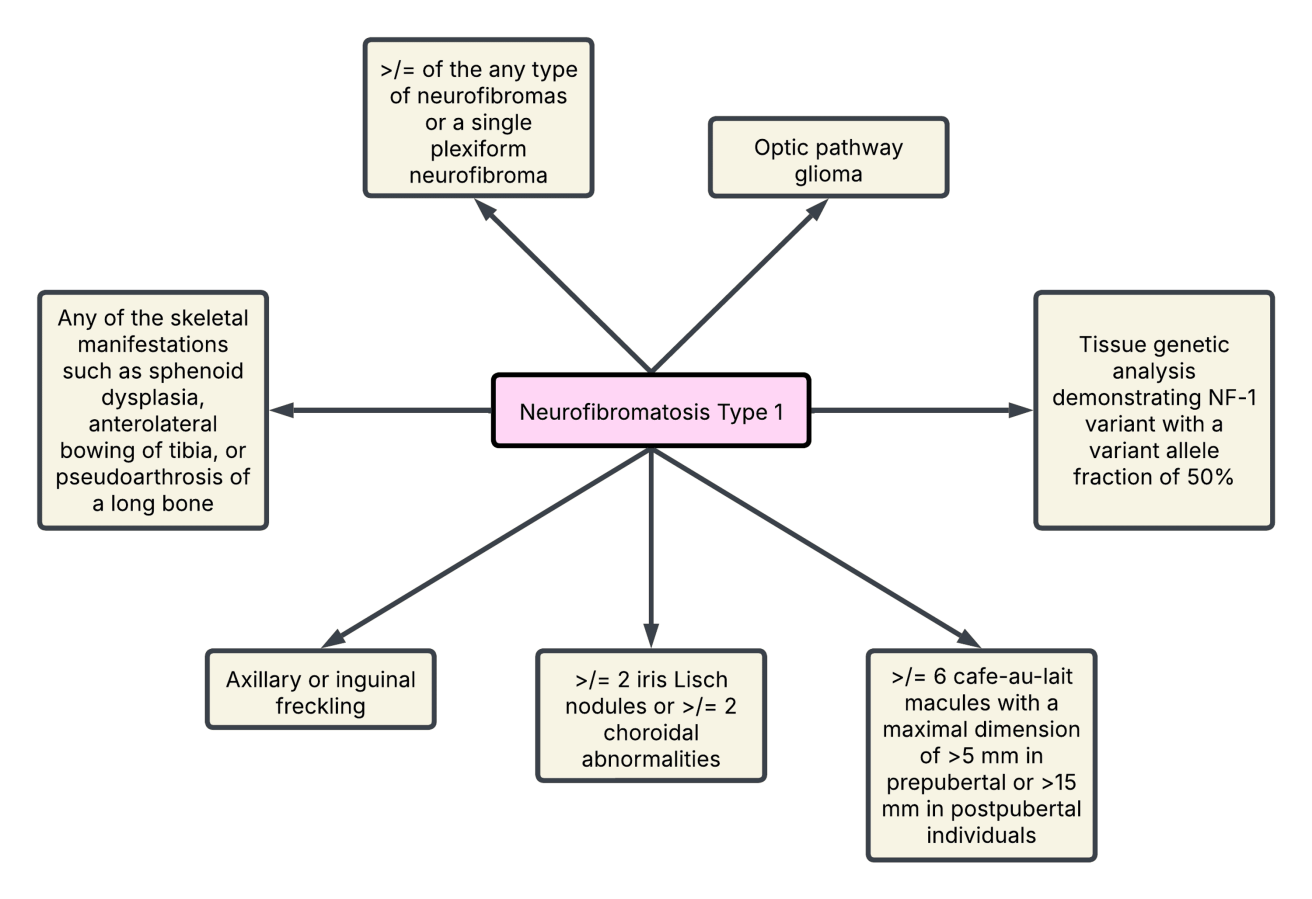
Diagnostic criteria for NF-1. A child with an NF-1-diagnosed parent requires one or more and a child without an NF-1-diagnosed parent requires two or more diagnostic criteria to merit a diagnosis of NF-1. NF-1: neurofibromatosis type 1 Figure created by the author based on an international consensus recommendation by Legius et al. [7].

NF-1 is an inherited disorder secondary to alterations in the NF-1 gene. NF-1 is a tumor suppressor gene and encodes for neurofibromin, a protein responsible for regulating cellular growth and differentiation. Mutation of the NF-1 gene may result in predisposition to NF-1, and the clinical manifestations depend on the type of mutation, age of the patient at which it occurs, and its association with other genetic alterations. The NF-1 gene is large in size, and it is difficult to identify the mutation site through genetic analysis. Hence, the diagnosis is based on clinical and imaging manifestations (Figure 6) [7].

Neurofibromas are benign peripheral nerve sheath tumors, arising from Schwann cells, most commonly associated with NF-1 and are seen in approximately 40-60% of patients. On the other hand, schwannomas, meningiomas, and ependymomas are the key manifestations of NF-2. Schwannoma as a NF-1 manifestation is rare [4]. Schwannoma along the gastrointestinal tract is an infrequent manifestation with the stomach being the most common location harboring around 83% of cases. Rectal schwannomas are exceptionally rare, and till date, very few cases have been reported in the literature [6,8-21]. A retrospective study by Bohlok et al. included 95 cases of colorectal schwannomas published in the literature, out of which 21 cases (21%) were involving the rectum [5]. An additional retrospective study by Gibson and Hornick included 26 colorectal schwannomas, out of which only three were involving the rectum [22]. None of the reported cases were associated with the NF-1 syndrome. Hence, we believe ours is the first case of rectal schwannoma associated with NF-1. 

Gastrointestinal schwannomas (GIS) are usually benign asymptomatic and identified incidentally as submucosal lesions on routine colonoscopy. If clinically symptomatic, patients manifest abdominal plain, hematochezia, constipation, tenesmus, or obstruction depending on the size of the mass. On CT, GIS has a heterogeneous appearance with variable enhancement. Due to high spatial resolution, MRI assists in identifying the close proximity location of the GIS to the nerves differentiating it from other GIMT. T2-weighted MR sequences demonstrate a target appearance of the tumor with peripheral high signal intensity (myxomatous tissue) and central low signal intensity (fibrocollagenous tissue) [23]. Histologically, the high signal intensity areas correspond to Antoni B where the fibers are loosely interspersed in myxomatous tissue, while low signal intensity areas correspond to Antoni B where the fibers are compactly arranged [5,23,24]. These tumors have heterogeneous signal intensity on T1-weighted MR sequences. With tumor progression and degeneration, these tumors are termed as ancient schwannomas and are characterized by cystic, hemorrhagic, or myxoid changes that can be seen as variable signal intensities on T1- and T2-weighted MR sequences. There may be peripheral enhancement of the solid tumor portion on contrast administration.

Grossly, GIS is a solid unencapsulated mass with a smooth gray or yellowish cut surface [25]. Hemorrhage, calcification, cystic changes, and necrosis are rare and help in differentiating GIS from gastrointestinal stromal tumors. Immunohistochemical staining is the gold standard diagnostic test for GIS. S-100 is a calcium-binding protein found abundant in Schwann cells and the tumors arising from them. Around 97% of GIS exhibit positive S-100 staining making it the most specific marker for diagnosis [26]. Although S-100 staining is also seen in neurofibromas, the density of positively stained cells is less as the neurofibromas are a mixture of mast cell, vascular elements, fibroblasts, and perineural-like cells [27], whereas schwannomas are solely composed of well-differentiated Schwann cells [27]. 

Surgical resection is the definitive treatment of benign schwannomas [5,28]. Histologic features and tumor size may aid in predicting tumor aggressiveness and recurrence risk [28]. Tumor with Kiel 67 (Ki-67) >5% is considered aggressive, while >10% is considered malignant. Mitotic index of >5 in a tumor of >5 cm size has high risk for metastases and/or recurrence [5]. However, using these parameters to classify whether the tumor is benign or malignant is still debatable [5]. Due to their low incidence, malignant transformation is rare and is reported in only 2% of cases [26,27].

## Conclusions

NF-1 is an autosomal dominant neurocutaneous disorder characterized by skin abnormalities, such as café-au-lait macules and skinfold freckling, as well as peripheral nerve sheath tumors such as neurofibromas, schwannomas, and various other tumors. Schwannomas, while less common in NF-1 than NF-2, do sometimes occur in NF-1 patients, most commonly along the cranial, spinal, or peripheral nerves. These tumors do, however, rarely occur in the gastrointestinal tract and even more rarely occur specifically in the rectum as demonstrated in our patient. Schwannoma must be considered in the differential for any rectal mass discovered in a patient with NF-1, so that the patient can be appropriately managed and treated.
